# Does the Australian Health Star Rating System Encourage Added Sugar Reformulation? Trends in Sweetener Use in Australia

**DOI:** 10.3390/nu13030898

**Published:** 2021-03-10

**Authors:** Cherie Russell, Sarah Dickie, Phillip Baker, Mark Lawrence

**Affiliations:** 1 School of Exercise and Nutrition Sciences, Deakin University, Geelong 3217, Australia; sdickie@deakin.edu.au (S.D.); phil.baker@deakin.edu.au (P.B.); mark.lawrence@deakin.edu.au (M.L.); 2 Institute for Physical Activity and Nutrition, Deakin University, Geelong 3217, Australia

**Keywords:** added sugar, health star rating, non-nutritive sweeteners, nutrient profiling, food policy, ultra-processed food

## Abstract

Dietary risk factors, including excess added sugar intake, are leading contributors to Australia’s burden of disease. An objective of the Australian Health Star Rating (HSR) system is to encourage the reformulation of packaged foods. Manufacturers may improve a product’s HSR by replacing added sugar with non-nutritive sweeteners (NNS). Concerns have been raised regarding the potential substitution effects of ultra-processed foods containing NNS for whole foods, and the long-term impact this may have on population health. The aim of this study was to determine whether the implementation of the HSR system has impacted the use of added sugars and NNS in the food supply. Four product categories were used: products with no added sweetener, products containing added sugar only, products containing NNS only, and products containing a combination of added sugar and NNS. Of 6477 newly released products analyzed displaying a HSR in Australia between 2014–2020, 63% contained added sugars. The proportion of new products sweetened with added sugars increased over time, while NNS use did not, despite a higher average and median HSR for products sweetened with NNS. These findings suggest that at the current time, the HSR system may not discourage the use of added sugars in new products or incentivize the reformulation of added sugar with NNS. As the health risks of NNS are questioned, increased reformulation of products with NNS to reduce the presence of added sugar in the food supply may not address broader health concerns. Instead, supporting the promotion of whole foods and drinks should be prioritized, as well as policy actions that reduce the proliferation and availability of UPFs.

## 1. Introduction

Dietary risk factors are among the leading contributors to Australia’s burden of disease [1]. This includes excess intake of added sugar, defined as caloric sweeteners added to foods during processing, preparation or at the table [2]. Frequent over-consumption of added sugar is associated with obesity, type-two diabetes, and dental caries, particularly from sugar-sweetened beverages (SSB) [3,4,5]. Despite the World Health Organization (WHO) recommendation to limit free sugar consumption (consisting of both added and naturally occurring sugars) to 10% of total energy intake [6], added sugar contributes 11-20% of energy intake in Australian diets [7].

The Healthy Food Partnership (the Partnership) was established in Australia in 2015 to address dietary risks associated with obesity and NCDs, including excess added sugar consumption [8]. Comprising stakeholders from industry, government, and public health, the Partnership aims to encourage healthy eating and incentivize food manufacturers to make ‘positive changes’ [8]. Despite a broad suite of potential policy actions available to reduce added sugar consumption and improve diet quality, including taxation of SSB, education and food environment interventions [9], the Partnership has focused predominantly on policies that promote nutrient reformulation, including voluntary targets for packaged, processed foods the Health Star Rating (HSR) labelling system and controlling the portion size of certain products [10]. Reformulation can be either overt, in which nutrient-profile changes are advertised to the consumer, or covert, in which compositional changes are not promoted [11].

Introduced in 2014, the HSR system is a voluntary, interpretive, front-of-pack labelling scheme designed to aid consumers in choosing ‘healthier’ packaged products within specific categories [12]. Products can display a rating from half a star (least healthy) to five stars (most healthy) in half star increments [13]. The number of stars is calculated based on a nutrient-profiling algorithm, which rewards so called ‘beneficial’ nutrients/ingredients (protein, fiber, and the percentage of fruit, vegetable, nut and legume content) and penalizes so-called ‘risk’ nutrients (energy, saturated fat, total sugar and sodium) [13]. The lack of transparency during the development of the algorithm has been criticized [14]. Other concerns raised in stakeholder submissions during a review of the system in mid-2017 include its leniency towards penalizing total sugar (despite an updated sugar penalty scale, which will be implemented by November 2022); the use of total sugar instead of added sugar; the possibility that the addition of positive nutrients/ingredients may be used to ‘game’ the system (increasing the star rating by adding synthetic ‘beneficial’ nutrients without subtracting ‘risk’ nutrients); and the exclusion of the level of processing in the algorithm [15]. Ultra-processed foods (UPF), categorized by the NOVA food processing classification system, are defined as industrial formulations which typically contain cosmetic and various other types of additives [16]. These products are designed to be hyper-palatable, affordable, convenient and are often marketed intensively [17]. A growing body of evidence has demonstrated a positive association between UPF intake and adverse health outcomes, including heart disease and type-two diabetes [17,18]. Previous research has demonstrated that the HSR does not distinguish between levels of processing, with 73% of newly released UPFs receiving a HSR of 2.5 or higher [19].

One approach manufacturers can use to improve a product’s HSR score is to reformulate a product by replacing its added sugar content (even partially) with a non-nutritive sweetener(s) (NNS) [20]. NNS are defined as non-caloric substances which impart sweetness when added to products [21] and are used in food reformulation to reduce the energy and added sugar content of foods while maintaining their palatability [22]. The association of NNS consumption with health outcomes is contested. Clinical trials have demonstrated a reduction in body mass index [23,24,25] and fasting blood glucose [26,27]. However, observational studies have reported associations between NNS consumption and weight gain [28,29,30,31], changes to the gut microbiome [32] and type-two diabetes [33,34]. Relationships from observational studies are correlational and may be biased, as participants with existing morbidities may be more likely to consume NNS than those without [9]. Concerns have also been raised regarding the potential substitution effects of UPF containing NNS for nutritious whole foods, and the long-term impact this may have on dietary balance and population health [35]. Reformulation of UPF with NNS may create a ‘health halo effect’, allowing industry to make these products appear ‘healthy’, potentially resulting in higher consumption of these foods [36], while also displacing nutritious foods from the diet.

Other factors that may contribute to the reformulation of products with NNS include the threat of government legislative action on products high in added sugar, increasing consumer demand for low sugar and low calorie products, and increasing consumer acceptance of ‘natural’ NNS (those that are naturally occurring in some form, such as steviol glycosides) [37,38,39]. Additionally, technological advances in NNS applications, driven by the development of new varieties of NNS, new extraction methods, and the ability to combine sweeteners to achieve desired sensory effects, has allowed the food industry to expand their use [40]. The sweetener industry is lucrative and expanding rapidly, with the global NNS market expected to grow from $2.3 billion in 2016 to $3.3 billion in 2022 [41]. In Australia, 12 NNS have been approved for use, and are permitted as table-top sweeteners and in many product categories including dairy, confectionary, bakery, cereal, processed fruits and vegetables and non-alcoholic beverages [42].

Currently, it is not known how the HSR system influences sweetener reformulation, including the extent to which NNS are used as replacements for added sugars; nor whether such reformulation improves the healthiness of the food supply, or instead provides a health halo for UPF. Thus, the aim of this study is to determine whether the implementation of the HSR system has impacted the use of added sugars and NNS in the food supply, and to compare the star ratings of sweetened products. In doing so, the study will address the following research questions:

- What are the trends of sweetener use in new products displaying a HSR label in the Australian food supply?
- What are the trends in added sugar and NNS content of reformulated products displaying a HSR?
- How does the HSR score differ between product categories sweetened with either added sugar or NNS?
- How does the level of processing differ between unsweetened products and those sweetened with added sugar and/or NNS?

## 2. Materials and Methods

### 2.1. Data Collection

Comprehensive, publicly available longitudinal databases assessing NNS use in Australia were not available for use at the time of the study. Thus, data were obtained from the Mintel Global New Product Database (GNPD) [43]. The GNPD captures all new and updated packaged food and beverage products released globally. All new Australian food and beverage products displaying a HSR between the 27th of June 2014 (the implementation date for the HSR system) and 30th June 2020 (the time of data collection) were included in the analysis. The Mintel ‘baby foods’ and ‘alcoholic drinks’ categories were excluded from the sample as they are not eligible to display a HSR. Information extracted included the product name, HSR, Mintel food category and sub-category, release date, product description, packaging images, nutrition composition, and ingredients list. For products sweetened with NNS, further information was collected, including previous iterations of the product and their sugar content.

### 2.2. Sweetener Classification

Four product categories were used for this study in relation to sweetener use: products with no added sweetener, products containing added sugar only, products containing NNS only, and products containing a combination of added sugar and NNS. NNS were categorized based on Food Standards Australia and New Zealand (FSANZ) classifications (where they are referred to as ‘intense sweeteners’) [42]. For the purposes of this study, sugar alcohols and other novel low-calorie sweeteners (including fructo- isomalto- and oligo-saccharides) were included in the category of NNS due to their negligible contribution to energy intake. As there is currently no Australian definition of added sugar, the United States Department of Agriculture classification was used for this study, in line with previous research [2,44,45]. Using this definition, some sugars which may be classified as ‘free sugars’ by other definitions were categorized as added sugar, including honey, some syrups and fruit concentrate [2]. Despite its multiple uses in food processing, maltodextrin was included as an added sugar, consistent with a similar analysis [45]. Single ingredient products, such as honey and table sugar, were excluded from the analysis as the sugar content of these products was not ‘added’.

### 2.3. Data Analysis

Duplicates (products released with new packaging but the same HSR/ingredients/nutrient content) were removed from the data. Data were coded using Microsoft Excel according to the number of health stars displayed, the category of sweetener used (added sugar, NNS, both or no sweetener), and the specific sweeteners added to each product (a full list is shown as Table 1). All products were classified by two researchers independently.

Products were also categorized by both the Australian Dietary Guidelines (ADG) [46] and the NOVA food-processing classification system [16]. Three coding categories were used to describe the alignment of foods with the ADGs: (i) ‘five-food group’(FFG) foods (fruit; vegetables; grains; meat, eggs, tofu, nuts, seeds, and legumes; milk, yoghurt, cheese, and alternatives; and mixed meals consisting mostly of FFG foods); (ii) Discretionary foods (foods and drinks without essential nutrients and high in saturated fats, sugars, salt and/or alcohol); and (iii) ‘other’ foods (culinary ingredients; formulated supplementary foods; and water). Further details of this classification scheme are published elsewhere [19]. The four NOVA food processing classification system categories were also applied: unprocessed or minimally processed foods; processed culinary ingredients; processed foods; and ultra-processed foods [16]. Statistical analysis was performed using Microsoft Excel and R statistical computing software. Descriptive statistics were generated for the data. This included the HSR averages, medians, and interquartile ranges (IQR) for each sweetener category. The number of products for each sweetener category, NOVA classification and ADG group were also calculated.

## 3. Results

### 3.1. Trends in Sweetener Use

Overall, 6477 new products were released with a HSR between June 2014 and June 2020. Of these products, 4213 contained at least one sweetener (65%) (Table 2). Added sugar was the most common sweetener used (63.5%), while NNS were present in 2.8% of products.

**Figure 1:**
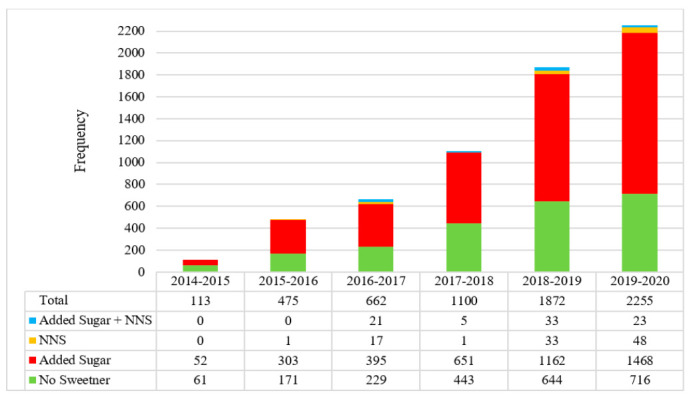
Products classified by sweetener type as a proportion of total new products released with a HSR each year since implementation (June 2014–June 2020).

The number of new products displaying a HSR increased each year after implementation (Figure 1). Additionally, the number of new products sweetened with added sugar and displaying a HSR has increased over time, both in quantity and as a proportion of the number of new products released to the market with a HSR (54% in 2014–2015, 66% in the 2019–2020) (Figure 1). The proportion of new products containing NNS remained approximately constant.

Stevia was the most common NNS in the sample, present in 91 products, or almost half of all products sweetened with NNS (*n* = 182). This was followed by sucralose (*n* = 54, 30 %) and acesulfame potassium (*n* = 35, 19%).

**Figure 2:**
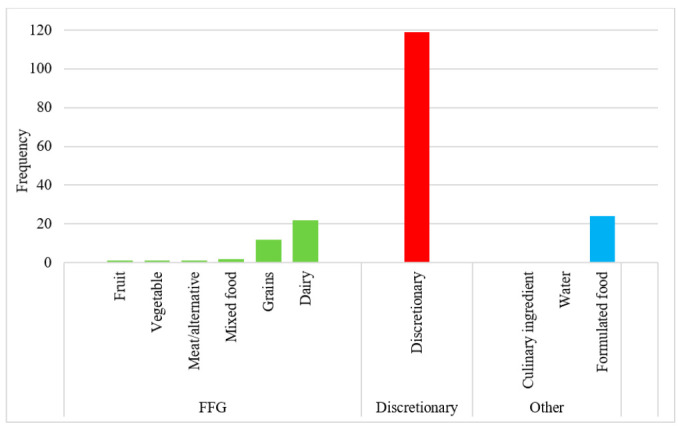
Frequency of sweetened HSR products by ADG category. FFG: Five Food Group

The ADG categories most likely to contain NNS (either alone or with added sugar) included discretionary items (*n* = 119, 65%), formulated food (*n* = 24, 13%) and FFG dairy products (*n* = 22, 12%) (Figure 2).

### 3.2. Trends in Added Sugar and Non-Nutritive Sweeteners Content Of Reformulated Products

Of the products sweetened with NNS, 11% (*n* = 20) were reformulated versions of previously released products. As added sugar is not listed on the nutrition information panel in Australia, the amount of added sugar present in a food product could not be quantified, and total sugar was used instead. The reformulated products had an average total sugar content 2.6 g/100 g (or mL/100mL) less than the previous versions. In many cases, products containing NNS were released alongside added-sugar products, as opposed to replacing existing products. Most of these foods were discretionary products (*n* = 10).

### 3.3. Difference in the Australian Health Star Rating of Sweetened Products

Products sweetened with added sugar had a lower average and median HSR than unsweetened products, or those sweetened exclusively with NNS (Table 3). For all products, except those sweetened with both NNS and added sugar, the HSR ranged from 0.5 to 5 stars.

### 3.4. Trends in Level of Processing of Sweetened Products

**Figure 3:**
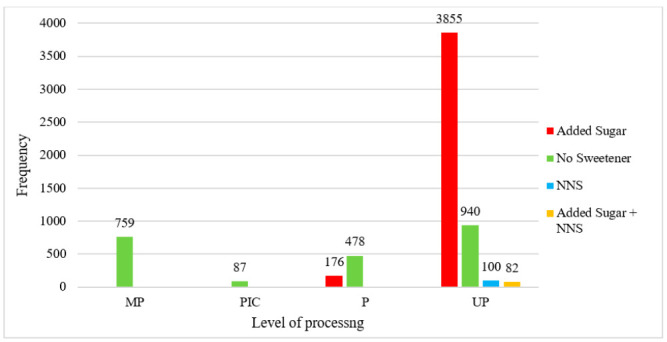
Frequency of sweetened HSR products by NOVA category. MP—Minimally processed; PCI—processed culinary ingredients; P—Processed; UP—Ultra-processed.

Over three quarters (76.8%) of products in the sample were classified as ultra-processed (Figure 3). In line with previous literature, all products sweetened with NNS (either alone or in combination with added sugar) were classified as ultra-processed [19]. Thus, the products in these categories were classified entirely as ultra-processed. Additionally, almost all products sweetened only with added sugar (95.6%) were categorized as ultra-processed. Comparatively, 41.5% of unsweetened products were classified as ultra-processed, while 33.5% were classified as minimally processed.

## 4. Discussion

The aim of this study was to determine whether the implementation of the HSR system has impacted the use of added sugar and NNS in new products entering the food supply, and to compare the star ratings of these products. The number of new products displaying a HSR has grown each year, suggesting an increasing uptake by manufacturers, consistent with the five-year review of the system [15]. However, the number and proportion of products sweetened with added sugar has also increased over time. Though total sugar and carbohydrates are listed on the nutrition information panel, the proportion of added sugar within the total sugar amount does not have to be declared under current Australian food regulations, thus the amount of this change could not be calculated. Comparatively, the rate of NNS use in new products with a HSR did not increase. Products containing NNS displayed a higher average and median HSR, despite all such products being classified as UP, and most also classified as a discretionary food.

These findings suggest that the HSR system may not incentivize the reformulation of added sugar with NNS or discourage the use of some added sugar in new products. As this study examined the frequency of new products containing sweeteners, the overall volume of added sugar within these products may have varied. This is an important area for future research. Though previous evaluations of the HSR system have shown a positive impact on reformulation, including small changes in energy, sodium and fiber [47,48,49], insignificant changes in added sugar have been reported in only one 2020 study [50]. The voluntary nature of the HSR system means that low product classifications do not have to be displayed, and therefore could be underrepresented in the market. This limits the comparability of products for consumers. This could also weaken the incentive effect of the HSR scheme for product reformulation when looking at the entire food supply. A similar proportion of products were sweetened with both added sugar and NNS, and NNS alone. Though only a small sample was available for analysis, this suggests that products are more likely to be reformulated with a combination of sweetener categories, rather than a direct replacement of sugar with NNS. Sugar serves multiple purposes in food, including texture and bulking properties, which may contribute to the partial substitution of sugar with alternative sweeteners [51].

Of all products in the sample, 63.5% were sweetened with added sugar, while only 2.8% were sweetened with NNS. This finding suggests that added sugars are still the preferred sweetener in the Australian marketplace. Though this is a lower percentage than can be seen in the food supply of other countries, including the United States (76%) [20], this still accounts for over two-thirds of all products that displayed a HSR. However, as the HSR system is voluntary, this finding does not represent the total proportion of sweetened products in the Australian food supply. In 2018, only 31% of eligible products displayed a HSR [15]. Due to the voluntary nature of the system, products with a lower score (such as those sweetened with added sugars) may be less likely to display the label, while reformulated products (such as those reformulated with NNS) may be more likely to be display the HSR, given the incentive provided by the system. Of concern is that the increasing number of products with added sugar in this sample may be indicative of increasing sweetness of the overall food supply, though this cannot be determined without information regarding of the volume of added sugar in each product [20]. When food sweetened with either added sugar or NNS is consumed routinely, especially earlier in life, this flavor profile becomes familiar and acceptable and can inform preferences for sweetened food [52]. Evidence suggests that this preference begins in utero, and continues over the lifespan [53]. Overstimulation of sweet taste receptors may limit tolerance for more complex, less sweet tastes, such as fruits and vegetables [54]. The potential impact that an increasingly sweet food supply may have on sweet preferences and liking, both in terms of added sugar and NNS, requires further investigation.

Previous research analyzing the added sugar and NNS content of the Australian food supply has relied on cross-sectional studies, thus a clear comparison with the results from this longitudinal study cannot be made. However, the combined results of the present sample aligns with some findings of a study by Probst et al. [55], who reported that in a sample of products from 2012, added sugar was found in 61% products [55], while only 31% of foods contained no added sweetener. However, the same study also reported that NNS were present in 68.8% of products. The accuracy of this particular finding is questionable as the study also reported that only 0.5% (*n* = 29) of the sample contained NNS [55]. Additionally, the sampling occurred prior to global recommendations to reduce the added sugar content of packaged foods and beverages [6].

Among a sample of products from the Australian food supply collected in 2015, Dunford et al. [56] reported that less than 1% contained NNS [56]. This finding was in contrast to Mexico, where NNS were found in 11% of products [56]. Mexico had introduced a tax on sugar-sweetened beverages (SSB) the previous year [56,57]. Recent research from Chile has also demonstrated the potential impact of regulation, including warning labels and advertising restrictions, on NNS presence in the food supply [58]. In a sample of 1,489 products, 815 (55.5%) contained NNS [58]. The large disparity between the Australian and Latin American context raises the question of whether the Australian marketplace has an inherent resistance to reformulation, or instead reflects a lack of penalties on added sugar to create sufficient incentive for reformulation. However, Mexico recently applied front of pack warning labels to products containing NNS, which may influence rates of use in the future [59]. Similar nutrient-profiling labelling systems exist globally, including ‘traffic light labelling’ in the United Kingdom, ‘Nutri-score’ in France, and ‘Keyhole’ labelling in Scandinavia. However, there is limited evaluation of product reformulation with NNS in these systems. More research is needed to determine whether an increase in NNS is influenced by policy actions to reduce added sugar, and to corroborate these findings in other contexts.

Stevia was the most prolific NNS used throughout the sample and was present in an increasing number of products each year. Stevia is considered a ‘natural sweetener’, which may provide a more distinct ‘health halo’ than other sweeteners, and thus may be more acceptable to consumers [38]. The rise in stevia use has also been reported in Chile [58]. A recent report produced by the Mintel Database also demonstrated increasing stevia use globally, particularly in Latin American countries where there are regulations related to added sugar [60].

Over three quarters (76.8%) of products in the sample were classified as UP. These results mirror the levels of processing found in new products with a HSR from Dickie et al. [19]. This finding suggests that the HSR system has not incentivized a reduction in the level of food processing, either explicitly or inadvertently. Interestingly, the levels of processing between products sweetened with NNS or added sugar were equivalent, despite the presence of NNS in a product automatically classifying that product as ultra-processed. Additionally, most products that contained NNS were classified as discretionary foods in accordance with the ADG classification system [61]. This may have masked the ‘health halo’ effects of the current HSR system application in practice, as a front-of-pack label applied to packaged, processed foods. Given the associations of both discretionary products and UPF with negative health outcomes [18,62], this indicates a significant public health concern for the future.

There is increasing attention on added sugar in Australia, with discussions around fiscal policy [63,64], educations campaigns [65,66] and added sugar labelling [67]. Often these policies target beverages, despite recent research suggesting that the contribution of added sugars to the food supply from SSBs is decreasing [68]. Comparatively, the sales volume of added sugars from UPF has increased, indicating a need to broaden policy actions beyond SSBs [68]. The findings of the present study may indicate that the current incentives for reformulation of added sugar in Australia may not be sufficiently aggressive. The leniency of the nutrient-profiling algorithm of the HSR in penalizing sugar has been widely criticized by public health experts [14,19,69]. Given the increased sugar penalties to be made mandatory in 2021, there may be an increased use of NNS and a decreased use of added sugar in future analyses of the HSR. However, as ‘added sugars’ are yet to replace ‘total sugars’ in the algorithm, these changes may not go far enough to encourage reformulation. Interestingly, there has been an increasing number of NNS receiving regulatory approval for use in Australia. This rising level of sweetener development and regulatory approval for their use in the food supply suggests that there may be a longer-term intention of manufacturing foods with these additives. Their current modest use, despite their increased availability for manufacturers, may reflect that current policy actions such as the HSR system do not provide sufficient incentive for their implementation in Australia. Stronger policy actions in the future may elicit a more substantial change in NNS levels in line with other countries. However, given the contested health risks of NNS, the classification of products containing NNS as UP, and the potential substitution effects of UPF containing NNS for nutritious whole foods, such reformulation may not address existing public health concerns. Instead, policy actions which address the profile of the broader food supply, including promoting nutritious whole foods and tackle the proliferation of UPF, should be prioritized.

### Strengths and Limitations

This study has a number of strengths. This was the first longitudinal study to demonstrate the changes in NNS use in the Australian context. This was also the first study to evaluate the impact of the HSR system on the reformulation of products containing any form of sweetener, as opposed to added sugar alone. With the increased consumption of NNS internationally [70,71,72,73,74,75,76,77,78,79,80], it is important that the addition of these sweeteners to the Australian food supply, and their subsequent intake, is monitored over time.

This study also has limitations. As reported in previous research [55,56], it was not possible to determine if the amount of added sugars or sweeteners in the food supply has changed over time, as quantified NNS and added sugar amounts are not listed on labels in Australia. A limitation of the Mintel GNPD is that only overtly reformulated products, i.e., those that have marketed a nutrient content claim, or otherwise advertised their reformulation, are included in the database. Thus, covertly reformulated products were not captured, potentially undermining the analysis. However, as consumer demand is one of the primary drivers of sugar reduction, manufacturers would thus be likely to advertise these compositional changes. As such, the number of products covertly sweetened with NNS is likely to be small. Other comprehensive, longitudinal databases assessing NNS use in Australia were limited. This is concerning, given the global trends of increasing NNS consumption and use in the food supply. Repetition of the present research using a broader and more comprehensive data in the future would strengthen the findings of this study.

## 5. Conclusions

Within the Australian food supply, an increasing number of new products released with a HSR contained added sugar, while the use of NNS has remained consistent over time. The HSR system may not incentivize the reformulation of added sugar with NNS or discourage the addition of any added sugar in new products. This is contrary to findings from countries with strong policy actions to reduce added sugar consumption. Most products displaying a HSR were discretionary foods and ultra-processed, particularly if they contained a sweetener. Products containing NNS received higher mean and median HSR scores than those sweetened with added sugar only, despite the low level of NNS use in the sample. While reformulating a product by replacing some level of added sugar with NNS may improve a product’s star rating, such actions would not address other influences on a product’s ‘healthiness’, including level of processing, and may inadvertently provide ultra-processed products with a health halo. These findings contrast with the increased number of regulatory approvals for NNS use in Australia, which may suggest the need for policy actions that create greater disincentives for added sugar use. With the ongoing contestations regarding the health risks of NNS, increased reformulation of products with NNS to reduce the presence of added sugar in the food supply may not address broader health concerns. Instead, supporting the promotion of whole foods and drinks should be prioritized, including policy actions that reduce the proliferation and availability of UPFs.

## Figures and Tables

**Table 1:** Add sugars and non-nutritive sweeteners included in the study.

Added Sugars	Non-Nutritive Sweeteners
Barley malt extract	Acesulphame Potassium
Corn syrup solids	Advantame
Dextrose	Alitame
Fructose	Aspartame
Fruit juice concentrate	Aspartame-acesulphame salt
Fruit puree concentrate	Cyclamate
Glucose	Erythritol
Glucose syrup solids	Fructo-oligosaccharide
Honey (honey, manuka honey)	Isomalto-oligosaccharide
Lactose	Isomalt
Maltodextrin	Malitol
Maltose	Mannitol
Molasses	Monk fruit extract
Nectar	Neotame
Powders (honey, fruit, agave, glucose)	Polydextrose
Sucrose	Saccharin
Starch hydrolysate	Sorbitol
Sugar (white, raw, brown, cane, icing, dusting, caster, coconut, palm, turbinado, cultured, fermented, demerara, inverted, caramelized, burnt, muscovado)	Steviol glycosides
Syrups (agave, brown rice, rice, fructose, glucose, sugar, invert sugar, golden, light corn, dark corn, high-fructose corn, maple, malt, sorghum, fruit, date, coconut, caramelized sugar, glucose-fructose, tapioca)	Sucralose
Treacle	Thaumatin
Trehalose	Xylitol

**Table 2:** Frequency and proportion of sweeteners used in new products that displayed a HSR between June 2014 and June 2020.

Sweetener Used	Frequency	Percentage of Sample
No sweetener	2264	35.0%
Add sugar	4031	62.2%
NNS	100	1.5%
Added sugar and NNS	82	1.3%

NNS: Non-nutritive Sweeteners.

**Table 3:** Frequency and proportion of sweeteners used in new products that displayed a HSR between June 2014 and June 2020.

Sweetener Used	HSR Average	HSR Median	HSR Range	HSR IQR
No sweetener	3.9	4.0	0.5–5	1.5
Add sugar	2.9	3.0	0.5–5	2
NNS	4.9	4.5	0.5–5	2
Added sugar and NNS	3.3	3.5	1.5–5	2.5

HSR: Health Star Rating; IQR: Interquartile Range.

## Data Availability

Restrictions apply to the availability of these data. Data was obtained from the Mintel Global New Product Database and are available from https://www.mintel.com/global-new-products-database (accessed on 11 February 2021) with the permission of Mintel.

## References

[1] Australian Institute of Health and Welfare Australian Burden of Disease Study 2015: Interactive Data on Risk Factor Burden. https://www.aihw.gov.au/reports/burden-of-disease/interactive-data-risk-factor-burden.

[2] Bowman S.A. (2017). Added sugars: Definition and estimation in the USDA Food Patterns Equivalents Databases. J. Food Compos. Anal..

[3] Hu F.B. (2013). Resolved: There is sufficient scientific evidence that decreasing sugar-sweetened beverage consumption will reduce the prevalence of obesity and obesity-related diseases. Obes. Rev..

[4] Morenga L.A.T., Howatson A.J., Jones R.M., Mann J. (2014). Dietary sugars and cardiometabolic risk: Systematic review and meta-analyses of randomized controlled trials of the effects on blood pressure and lipids. Am. J. Clin. Nutr..

[5] Scientific Advisory Committee on Nutrition (2015). Carbohyrates and Health.

[6] World Health Organization (2017). Sugars Intake for Adults and Children: Guideline. 2015.

[7] Lei L., Rangan A., Flood V.M., Louie J.C.Y. (2016). Dietary intake and food sources of added sugar in the Australian population. Br. J. Nutr..

[8] Healthy Food Partnership-About the Partnership. https://www1.health.gov.au/internet/main/publishing.nsf/Content/about-the-partnership.

[9] Russell C., Grimes C., Baker P., Sievert K., Lawrence M.A. (2020). The drivers, trends and dietary impacts of non-nutritive sweeteners in the food supply: A narrative review. Nutr. Res. Rev..

[10] Scrinis G. (2015). Reformulation, fortification and functionalization: Big Food corporations’ nutritional engineering and marketing strategies. J. Peasant. Stud..

[11] Kushi L.H., Nestle M. (2003). Food Politics: How the Food Industry Influences Nutrition and Health. J. Public Health Policy.

[12] Food Regulation Secretariat Legislative and Governance Forum on Food Regulation Communique 27 June 2014. https://foodregulation.gov.au/internet/fr/publishing.nsf/Content/forum-communique-2014-June.

[13] Health Star Rating System Calculator and Artwork. http://www.healthstarrating.gov.au/internet/healthstarrating/publishing.nsf/Content/calculator.

[14] Lawrence M.A., Dickie S., Woods J.L. (2018). Do Nutrient-Based Front-of-Pack Labelling Schemes Support or Undermine Food-Based Dietary Guideline Recommendations? Lessons from the Australian Health Star Rating System. Nutrients.

[15] MP Consulting. Health Star Rating System—Five Year Review Report. http://www.healthstarrating.gov.au/internet/healthstarrating/publishing.nsf/Content/D1562AA78A574853CA2581BD00828751/$File/Health-Star-Rating-System-Five-Year-Review-Report.pdf.

[16] Monteiro C.A., Cannon G., Levy R., Moubarac J.-C., Jaime P., Martins A.P., Canella D., Louzada M., Parra D. (2016). NOVA. The star shines bright. World Nutr..

[17] Monteiro C.A., Cannon G., Levy R.B., Moubarac J.-C., Louzada M.L., Rauber F., Khandpur N., Cediel G., Neri D., Martinez-Steele E. (2019). Ultra-processed foods: What they are and how to identify them. Public Health Nutr..

[18] Elizabeth L., Machado P., Zinöcker M., Baker P., Lawrence M. (2020). Ultra-Processed Foods and Health Outcomes: A Narrative Review. Nutrients.

[19] Dickie S., Woods J.L., Baker P., Elizabeth L., Lawrence M.A. (2020). Evaluating Nutrient-Based Indices against Food- and Diet-Based Indices to Assess the Health Potential of Foods: How Does the Australian Health Star Rating System Perform after Five Years?. Nutrients.

[20] Popkin B.M., Hawkes C. (2016). Sweetening of the global diet, particularly beverages: Patterns, trends, and policy responses. Lancet Diabetes Endocrinol..

[21] Joint FAO and WHO Expert Committee on Food Additives (1968). Specifications for the Identity and Purity of Food Additives and their Toxicological Evaluation: Some Flavouring Substances and Non-Nutritive Sweetening Agents.

[22] Chattopadhyay S., Raychaudhuri U., Chakraborty R. (2014). Artificial sweeteners—A review. J. Food Sci. Technol..

[23] Peters J.C., Wyatt H.R., Foster G.D., Pan Z., Wojtanowski A.C., Veur S.S.V., Herring S.J., Brill C., Hill J.O. (2014). The effects of water and non-nutritive sweetened beverages on weight loss during a 12-week weight loss treatment program. Obesity.

[24] Blackburn G.L., Kanders B.S., Lavin P.T., Keller S.D., Whatley J. (1997). The effect of aspartame as part of a multidisciplinary weight-control program on short- and long-term control of body weight. Am. J. Clin. Nutr..

[25] Imamura F., O’Connor L., Ye Z., Mursu J., Hayashino Y., Bhupathiraju S.N., Forouhi N.G. (2015). Consumption of sugar sweetened beverages, artificially sweetened beverages, and fruit juice and incidence of type 2 diabetes: Systematic review, meta-analysis, and estimation of population attributable fraction. BMJ.

[26] Raben A., Vasilaras T.H., Møller A.C., Astrup A. (2002). Sucrose compared with artificial sweeteners: Different effects on ad libitum food intake and body weight after 10 wk of supplementation in overweight subjects. Am. J. Clin. Nutr..

[27] Nichol A.D., Holle M.J., An R. (2018). Glycemic impact of non-nutritive sweeteners: A systematic review and meta-analysis of randomized controlled trials. Eur. J. Clin. Nutr..

[28] Toews I., Lohner S., De Gaudry D.K., Sommer H., Meerpohl J.J. (2019). Association between intake of non-sugar sweeteners and health outcomes: Systematic review and meta-analyses of randomised and non-randomised controlled trials and observational studies. BMJ.

[29] Fowler S.P., Williams K., Resendez R.G., Hunt K.J., Hazuda H.P., Stern M.P. (2008). Fueling the Obesity Epidemic? Artificially Sweetened Beverage Use and Long-term Weight Gain. Obesity.

[30] Miller P.E., Perez V. (2014). Low-calorie sweeteners and body weight and composition: A meta-analysis of randomized controlled trials and prospective cohort studies. Am. J. Clin. Nutr..

[31] Stellman S.D., Garfinkel L. (1986). Artificial sweetener use and one-year weight change among women. Prev. Med..

[32] Ruiz-Ojeda F.J., Plaza-Díaz J., Sáez-Lara M.J., Gil A. (2019). Effects of sweeteners on the gut microbiota: A review of experimental studies and clinical trials. Adv. Nutr..

[33] O’Connor L., Imamura F., Lentjes M.A.H., Khaw K.-T., Wareham N.J., Forouhi N.G. (2015). Prospective associations and population impact of sweet beverage intake and type 2 diabetes, and effects of substitutions with alternative beverages. Diabetologia.

[34] Bhupathiraju S.N., Pan A., Malik V.S., Manson J.E., Willett W.C., van Dam R.M., Hu F.B. (2013). Caffeinated and caffeine-free beverages and risk of type 2 diabetes. Am. J. Clin. Nutr..

[35] Sylvetsky A.C., Hiedacavage A., Shah N., Pokorney P., Baldauf S., Merrigan K., Smith V., Long M.W., Black R., Robien K. (2019). From biology to behavior: A cross-disciplinary seminar series surrounding added sugar and low-calorie sweetener consumption. Obes. Sci. Pr..

[36] Brownell K.D., Koplan J.P. (2011). Front-of-Package Nutrition Labeling—An Abuse of Trust by the Food Industry?. N. Engl. J. Med..

[37] Bearth A., Cousin M.-E., Siegrist M. (2014). The consumer’s perception of artificial food additives: Influences on acceptance, risk and benefit perceptions. Food Qual. Preference.

[38] Kamarulzaman N.H., Jamal K., Vijayan G., Jalil S.M.A. (2014). Will Consumers Purchase Stevia as a Sugar Substitute?: An Exploratory Study on Consumer Acceptance. J. Food Prod. Mark..

[39] Gardner C., Wylie-Rosett J., Gidding S.S., Steffen L.M., Johnson R.K., Reader D., Lichtenstein A.H. (2012). Nonnutritive Sweeteners: Current Use and Health Perspectives: A Scientific Statement from the American Heart Association and the American Diabetes Association. Diabetes Care.

[40] Bakal A.I. (2001). Chapter 26: Mixed Sweetener Functionality. Food Science and Technology.

[41] (2020). Global M&A Partners. Food Ingredients: Adding Zest to the Food and Beverage Industry. https://www.bglco.com/wp-content/uploads/2018/05/GMAP-Food-and-Beverage-Newsletter_Food_Ingredients_1.29.20-Update-to-5.17.18-1.pdf.

[42] Food Standards Australia New Zealand Intense Sweeteners. https://www.foodstandards.gov.au/consumer/additives/Pages/Sweeteners.aspx.

[43] Mintel Global New Products Database (GNDP). https://www.mintel.com/global-new-products-database.

[44] United States Department of Agriculture (2009). USDA Database for the Added Sugars Content of Selected Foods, Release 1. http://www.ars.usda.gov/services/docs.htm?docid=12107.

[45] Louie J.C.Y., Moshtaghian H., Boylan S., Flood V.M., Rangan A.M., Barclay A.W., Brandmiller J.C., Gill T.P. (2015). A systematic methodology to estimate added sugar content of foods. Eur. J. Clin. Nutr..

[46] National Health and Medical Research Council Australian Dietary Guidelines 2013. https://www.eatforhealth.gov.au/sites/default/files/files/the_guidelines/n55_australian_dietary_guidelines.pdf.

[47] Morrison H., Meloncelli N., Pelly F.E. (2019). Nutritional quality and reformulation of a selection of children’s packaged foods available in Australian supermarkets: Has the Health Star Rating had an impact?. Nutr. Diet..

[48] Herrera A.M.M., Crino M., Erskine H.E., Sacks G., Ananthapavan J., Ni Mhurchu C., Lee Y.Y. (2018). Cost-Effectiveness of Product Reformulation in Response to the Health Star Rating Food Labelling System in Australia. Nutrients.

[49] Mhurchu C.N., Eyles H., Choi Y.-H. (2017). Effects of a voluntary front-of-pack nutrition labelling system on packaged food reformulation: The health star rating system in New Zealand. Nutrients.

[50] Bablani L., Ni Mhurchu C., Neal B., Skeels C.L., Staub K.E., Blakely T. (2020). The impact of voluntary front-of-pack nutrition labelling on packaged food reformulation: A difference-in-differences analysis of the Australasian Health Star Rating scheme. PLoS Med..

[51] Koivistoinen P., Hyvönen L. (1985). The use of sugar in foods. Int. Dent. J..

[52] Sullivan S.A., Birch L.L. (1990). Pass the sugar, pass the salt: Experience dictates preference. Dev. Psychol..

[53] Ventura A.K., Worobey J. (2013). Early Influences on the Development of Food Preferences. Curr. Biol..

[54] Benton D. (2010). The plausibility of sugar addiction and its role in obesity and eating disorders. Clin. Nutr..

[55] Probst Y.C., Dengate A., Jacobs J., Louie J.C., Dunford E.K. (2017). The major types of added sugars and non-nutritive sweeteners in a sample of Australian packaged foods. Public Health Nutr..

[56] Dunford E.K., Taillie L.S., Miles D.R., Eyles H., Tolentino-Mayo L., Ng S.W. (2018). Non-Nutritive Sweeteners in the Packaged Food Supply—An Assessment across 4 Countries. Nutrients.

[57] Colchero M.A., Rivera-Dommarco J., Popkin B.M., Ng S.W. (2017). In Mexico, Evidence Of Sustained Consumer Response Two Years After Implementing A Sugar-Sweetened Beverage Tax. Health Aff..

[58] Sambra V., López-Arana S., Cáceres P., Abrigo K., Collinao J., Espinoza A., Valenzuela S., Carvajal B., Prado G., Peralta R. (2020). Overuse of Non-caloric Sweeteners in Foods and Beverages in Chile: A Threat to Consumers’ Free Choice?. Front. Nutr..

[59] White M., Barquera S. (2020). Mexico Adopts Food Warning Labels, Why Now?. Health Syst. Reform.

[60] Mattucci S. Ingredient Watch: Stevia. https://clients.mintel.com/insight/ingredient-watch-stevia?fromSearch=%3Ffreetext%3Dstevia.

[61] Health Star Rating System Guidance for Industry: Calculator and Style Guide. http://www.healthstarrating.gov.au/internet/healthstarrating/publishing.nsf/Content/E380CCCA07E1E42FCA257DA500196044/$File/Health-Star-Rating-system-Calculator-and-Style-Guide.pdf.

[62] Monteiro C.A., Cannon G., Lawrence M., Costa Louzada M.d., Pereira Machado P. (2019). Ultra-Processed Foods, Diet Quality, and Health Using the NOVA Classification System.

[63] Allen W.M.K., Allen K.J. (2019). Should Australia tax sugar-sweetened beverages?. J. Paediatr. Child Health.

[64] Cobiac L.J., Tam K., Veerman L., Blakely T. (2017). Taxes and Subsidies for Improving Diet and Population Health in Australia: A Cost-Effectiveness Modelling Study. PLoS Med..

[65] Morley B., Niven P., Dixon H., Swanson M., Szybiak M., Shilton T., Pratt I., Slevin T., Hill D., Wakefield M. (2016). Population-based evaluation of the ‘LiveLighter’ healthy weight and lifestyle mass media campaign. Health Educ. Res..

[66] Cook R. Rethink Sugary Drink–Building A Powerful, Sustainable Campaign on A Shoestring. Proceedings of the International Social Marketing Conference 2016 Societal Wellbeing.

[67] Food Regulation Standing Committee (2019). Policy Paper: Labelling of Sugars on Packaged Foods and Drinks.

[68] Baker P., Machado P., Santos T., Sievert K., Backholer K., Hadjikakou M., Russell C., Huse O., Bell C., Scrinis G. (2020). Ultra-processed foods and the nutrition transition: Global, regional and national trends, food systems transformations and political economy drivers. Obes. Rev..

[69] Peters S.A.E., Dunford E., Jones A., Ni Mhurchu C., Crino M., Taylor F., Woodward M., Neal B. (2017). Incorporating Added Sugar Improves the Performance of the Health Star Rating Front-of-Pack Labelling System in Australia. Nutrients.

[70] Sylvetsky A.C., Welsh J.A., Brown R.J., Vos M.B. (2012). Low-calorie sweetener consumption is increasing in the United States. Am. J. Clin. Nutr..

[71] (2014). Consumption of non-nutritive sweeteners and nutritional status in 10-16 year old students. Arch. Argent. Pediatr..

[72] Bleich S.N., Wolfson J.A., Vine S., Wang Y.C. (2014). Diet-Beverage Consumption and Caloric Intake Among US Adults, Overall and by Body Weight. Am. J. Public Health.

[73] Bolt-Evensen K., Vik F.N., Stea T.H., Klepp K.-I., Bere E. (2018). Consumption of sugar-sweetened beverages and artificially sweetened beverages from childhood to adulthood in relation to socioeconomic status—15 years follow-up in Norway. Int. J. Behav. Nutr. Phys. Act..

[74] Drewnowski A., Rehm C.D. (2015). Socio-demographic correlates and trends in low-calorie sweetener use among adults in the United States from 1999 to 2008. Eur. J. Clin. Nutr..

[75] Mesirow M.S., Welsh J.A. (2015). Changing Beverage Consumption Patterns Have Resulted in Fewer Liquid Calories in the Diets of US Children: National Health and Nutrition Examination Survey 2001–2010. J. Acad. Nutr. Diet..

[76] Mattes R.D., Popkin B.M. (2008). Nonnutritive sweetener consumption in humans: Effects on appetite and food intake and their putative mechanisms. Am. J. Clin. Nutr..

[77] Ng S.W., Slining M.M., Popkin B.M. (2012). Use of Caloric and Noncaloric Sweeteners in US Consumer Packaged Foods, 2005–2009. J. Acad. Nutr. Diet..

[78] Piernas C., Ng S.W., Popkin B. (2013). Trends in purchases and intake of foods and beverages containing caloric and low-calorie sweeteners over the last decade in the United States. Pediatr. Obes..

[79] Sylvetsky A.C., Jin Y., Clark E.J., Welsh J.A., Rother K.I., Talegawkar S.A. (2017). Consumption of Low-Calorie Sweeteners among Children and Adults in the United States. J. Acad. Nutr. Diet..

[80] Fakhouri T.H., Kit B.K., Ogden C.L. (2012). Consumption of Diet Drinks in the United States, 2009–2010. NCHS Data Brief. https://pubmed.ncbi.nlm.nih.gov/23102235/.

